# On the Potential of Gallium- and Indium-Based Liquid Metal Membranes for Hydrogen Separation

**DOI:** 10.3390/membranes12010075

**Published:** 2022-01-07

**Authors:** Leon R. S. Rosseau, José A. Medrano, Rajat Bhardwaj, Earl L. V. Goetheer, Ivo A. W. Filot, Fausto Gallucci, Martin van Sint Annaland

**Affiliations:** 1 Department of Chemical Engineering and Chemistry, Eindhoven University of Technology, P.O. Box 513, 5600 MB Eindhoven, The Netherlands; L.R.S.Rosseau@tue.nl (L.R.S.R.); J.A.Medrano.Jimenez@tue.nl (J.A.M.); I.A.W.Filot@tue.nl (I.A.W.F.); F.Gallucci@tue.nl (F.G.); 2 Department of Sustainable Process and Energy Systems, Nederlandse Organisatie Voor Toegepast-Natuurwetenschappelijk Onderzoek, Leeghwaterstraat 44, 2628 CA Delft, The Netherlands; rajat.bhardwaj@tno.nl (R.B.); earl.goetheer@tno.nl (E.L.V.G.)

**Keywords:** dense metal membrane, microkinetics, Sieverts’ law, hydrogen, liquid metals

## Abstract

The concept of liquid metal membranes for hydrogen separation, based on gallium or indium, was recently introduced as an alternative to conventional palladium-based membranes. The potential of this class of gas separation materials was mainly attributed to the promise of higher hydrogen diffusivity. The postulated improvements are only beneficial to the flux if diffusion through the membrane is the rate-determining step in the permeation sequence. Whilst this is a valid assumption for hydrogen transport through palladium-based membranes, the relatively low adsorption energy of hydrogen on both liquid metals suggests that other phenomena may be relevant. In the current study, a microkinetic modeling approach is used to enable simulations based on a five-step permeation mechanism. The calculation results show that for the liquid metal membranes, the flux is limited by the dissociative adsorption over a large temperature range, and that the membrane flux is expected to be orders of magnitude lower compared to the membrane flux through pure palladium membranes. Even when accounting for the lower cost of the liquid metals compared to palladium, the latter still outperforms both gallium and indium in all realistic scenarios, in part due to the practical difficulties associated with making liquid metal thin films.

## 1. Introduction

Hydrogen selective membranes have the potential to enable efficient and low-cost hydrogen separation and purification. Dense membranes, often based on palladium, have specifically attracted a lot of attention in this area, as they feature a relatively high flux alongside a high selectivity towards hydrogen. The relatively small size of the interstitial sites through which the permeating species should diffuse, along with favourable activity for the dissociation of hydrogen compared to other molecules enables this high selectivity for hydrogen [1,2]. Many alternative metals have been proposed, but palladium continues to be the metal of choice in this regard, as its catalytic activity and observed hydrogen flux have not been matched by other metals [3,4]. The alloying of palladium with metals such as gold, silver and copper has been studied in detail as this allows for an increase in permeability (in case of silver at specific concentrations), stability and chemical resistance of the membrane (in case of copper and gold), increasing the potential for the application of membrane separators in cost-effective production of ultra-pure hydrogen [5,6,7].

Recently, the concept of liquid metal membranes was introduced, adding to the growing collection of non-palladium-based hydrogen selective membranes [8]. It is hypothesized that an increase of the membrane flux is possible, provided that the membrane operates in the diffusion-limited regime. In this regime, the diffusivity and solubility govern the flux and the former parameter is inherently higher in the metallic liquid phase as compared to a solid membrane of the same metal [8]. Additionally, a broader operating window is expected as a liquid metal should not suffer from embrittlement or other common forms of solid membrane deterioration [9,10,11]. On the basis of their low melting points and large liquid temperature ranges, gallium and indium were identified as candidate liquid metals by Datta et al. [12]. In their study, it was measured that the permeability of a gallium-based membrane is 35 times higher than that of a palladium membrane. However, it was also reported that the high surface tension of the liquid metals and complex wetting behavior introduces practical complications in the production of thin films and in the selection of an appropriate support material. These complications were addressed by the use of a sandwiched configuration with SiC porous supports and rather thick films (in the order of several hundred μm) [8]. Hence, whilst the permeability is much higher compared to palladium, the flux in actual application is expected to be lower as palladium-based membranes with a thickness of several μm can be prepared.

Despite this, the initial performance of the liquid metal membrane concept justifies further investigations to elucidate the fundamental advantages of the concept and provide a basis for optimization of these novel separators. In the current paper, the transport of hydrogen through gallium- and indium-based liquid metal membranes will be modeled and compared to palladium-based solid membranes in an effort to assess which of the liquid metals is most suited for hydrogen separation and to investigate the possibilities for increased performance by means of a rate-determining step analysis.

**Figure 1:**
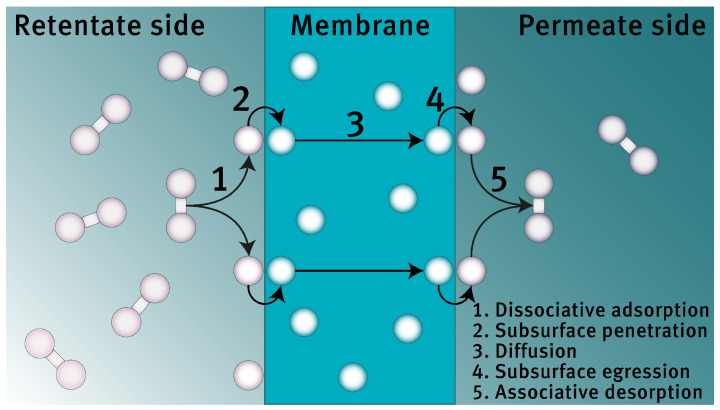
Schematic representation of the five-step permeation mechanism.

To gain the required fundamental understanding of the processes and phenomena involved, a quantitative description of the membrane flux is essential. It is generally accepted that transport of hydrogen through a dense membrane occurs through the solution-diffusion mechanism, which features five steps (excluding transport through the support material and bulk transport in the gas phase on either side of the membrane), as shown schematically in Figure 1 [13]. Initial studies based on this mechanism were already undertaken by Deveau et al. and Yen et al. (from the same research group) and the solubility and diffusivity of hydrogen in gallium and indium were estimated both through experiments and modeling efforts [14,15]. The modeling study relies on the simplicity of Sieverts’ law for predictions regarding the flux [13]. This rather simple description of the permeation behavior, as shown in Equation (1), was rationalized by assuming that diffusion through the membrane is the rate-determining step. For palladium-based membranes, this assumption is valid in situations with rather thick membranes, high temperatures and low hydrogen pressures, which is basically the case in all current applications [13,16,17,18]. For the proposed liquid metal membranes however, it cannot a priori be assumed that this approximation holds. Due the relatively low binding energy of hydrogen on gallium and indium, compared to palladium, it can be expected that a significant energetic barrier is present for the dissociation of hydrogen [15,19]. This energetic barrier lowers the rate of dissociation, which may be so slow that the overall permeation rate is no longer governed by the rate of diffusion.

(1) $$J_{H2}=\frac{D_{H}K}{2\delta }\left(p_{H2,r}^{0.5}-p_{H2,p}^{0.5}\right)$$

In absence of a sound justification that Sieverts’ law can be directly applied, a more fundamental approach is required wherein all elementary reaction steps describing the kinetic and diffusion events are treated and wherein no approximation on a possible rate-determining step is invoked a priori. A microkinetic model built on these grounds enables the optimization of existing membranes by means of a sensitivity analysis. Such models have been published in previous works, and will be appended for use in the current study to allow for thorough analysis of the performance of gallium- and indium-based liquid metal membranes.

This paper is organized as follows. First, the model is introduced and the main assumptions are outlined. Next, the model is validated based on experimental findings for solid palladium membranes. Thereafter, predictions regarding liquid metal membranes are presented, along with a validation of the calculated diffusion coefficient. The most salient finding of this study is the fact that, contrary to what was assumed in earlier works, the dissociative adsorption of hydrogen is the slowest step in the permeation mechanism in most cases. Due to this, the high diffusion coefficient of hydrogen in the liquid phase cannot be exploited and the performance of liquid metal membranes based on gallium or indium will not exceed that of the conventional palladium membrane in any realistic scenario.

## 2. Model Formulation

The microkinetic model employed in this study builds upon the work of Yen et al., which in turn is based on the work by Ward and Dao [13,15,20]. The most salient feature of the model proposed by Yen et al. is that it requires minimal need for empirical parameters [21]. In the current paper, the microkinetic model is set up in a general fashion, so that it allows for the description of the permeation behavior of both liquid and solid dense membranes. This attribute will later be used for model validation, as there is an abundance of experimental data for palladium-based membranes with which the predictions of this model may be compared.

As mentioned in the introduction, it is generally accepted that hydrogen is transported through a dense membrane in the following sequential steps (also see Figure 1) [13,15,22].

1. Gas-phase diffusion of molecular hydrogen from the retentate bulk to the membrane surface.
2. Dissociative adsorption of molecular hydrogen on the membrane surface at the retentate side.
3. Subsurface penetration of atomic hydrogen.
4. Diffusion of atomic hydrogen from the retentate side to the permeate side of the membrane.
5. Subsurface egression of atomic hydrogen.
6. Associative desorption of molecular hydrogen on the membrane surface at the permeate side.
7. Gas-phase diffusion of molecular hydrogen from the membrane surface to the permeate bulk.

The objective of this study is to look into the differences between palladium-based membranes and liquid metal membranes and to be able to provide a basis for optimization of the latter. This means that optimization of the separation module, to minimize external mass transfer resistances, is out of scope of the current paper. With this in mind, the first and the last step will be left out of consideration (as well as transport through the porous support), yielding the elementary reaction steps shown in Table 1.

It can be seen that steps 4 and 5 are the permeate-side counterparts of steps 2 and 1, respectively. This means that usually, these steps do not need to be treated separately; their kinetic description may be integrated in the description of their retentate-side counterparts [13]. In the current model, however, rate constants for surface processes depend on surface coverage, which differs for both surfaces and thus requires separate consideration.

Following the analogy of Deveau et al., the activity coefficients were set to unity by which the rates of the different elementary reaction steps can be expressed as a function of surface coverage and concentration, as shown in Equations (2)–(6) [20,23]. It should be mentioned that herein the effect of (macroscopic) surface roughness is neglected as data on the surface roughness is often undocumented in membrane-related publications.

(2) $$\begin{array}{cc} r_{1} & =c_{s}\left(k_{1}^{+}\frac{p_{H_{2,r}}}{p^{0}}{\left(1-\theta_{r}\right)}^{2}-k_{1}^{-}\theta_{r}^{2}\right) \end{array}$$

(3) $$\begin{array}{cc} r_{2} & =c_{s}c_{b}\chi_{H}\;\cdot \;M{}_{s}\left(k_{2}^{+}\theta_{r}\left(1-x_{r}\right)-k_{2}^{-}\left(1-\theta_{r}\right)x_{r}\right) \end{array}$$

(4) $$\begin{array}{cc} r_{3} & =\frac{D_{H}c_{b}\chi_{H}\;\cdot \;M{}_{s}}{\delta }\left(x_{r}\left(1-x_{p}\right)-x_{p}\left(1-x_{r}\right)\right) \end{array}$$

(5) $$\begin{array}{cc} r_{4} & =c_{s}c_{b}\chi_{H}\;\cdot \;M{}_{s}\left(k_{2}^{-}\left(1-\theta_{p}\right)x_{p}-k_{4}^{-}\theta_{p}\left(1-x_{p}\right)\right) \end{array}$$

(6) $$\begin{array}{cc} r_{5} & =c_{s}\left(k_{5}^{+}\theta_{p}^{2}-k_{1}^{+}\frac{p_{H_{2,p}}}{p^{0}}{\left(1-\theta_{p}\right)}^{2}\right) \end{array}$$

Equations (2)–(6) require the rate constants and the diffusion coefficient to enable a quantitative description of the permeation behavior. The microkinetic approach adopted here allows for the expression of these parameters as Arrhenius equations, as shown in Equations (7)–(9) [23]. Through transition state theory, the corresponding pre-exponential factors can be described via Equations (10) and (11) [24].

It should be noted that the diffusion coefficient of a solute in a liquid is commonly calculated through the Stokes–Einstein equation, being a less complicated approach. The diffusion coefficient was calculated in this way for liquid gallium and liquid indium, but the obtained values differed three orders of magnitude from experimental data and hence this approach was abandoned and Equation (9) was adopted instead [14,25]. A reason for the observed non-validity may be that the viscosity and density of liquid metals are unusually high and that the Stokes–Einstein equation generally holds for larger molecules rather than small atoms as solute [25]. The fact that the Stokes–Einstein equation largely underestimates the diffusion coefficient of hydrogen in liquid metals was also observed by Jaske and Pasturel before [26].

(7) $$\begin{array}{cc} k_{i}^{+} & =\Lambda_{i}^{+}\exp\left(-\frac{E_{i}^{+}}{RT}\right) \end{array}$$

(8) $$\begin{array}{cc} k_{i}^{-} & =\Lambda_{i}^{-}\exp\left(-\frac{E_{i}^{-}}{RT}\right) \end{array}$$

(9) $$\begin{array}{cc} D_{H} & =\Lambda_{D}\exp\left(-\frac{E_{D}}{RT}\right) \end{array}$$

(10) $$\begin{array}{cc} \Lambda_{i}^{+} & =\frac{k_{B}T}{h}\exp\left(\frac{\Delta S_{i}^{+,‡}}{R}\right) \end{array}$$

(11) $$\begin{array}{cc} \Lambda_{i}^{-} & =\frac{k_{B}T}{h}\exp\left(\frac{\Delta S_{i}^{-,‡}}{R}\right) \end{array}$$

**Figure 2:**
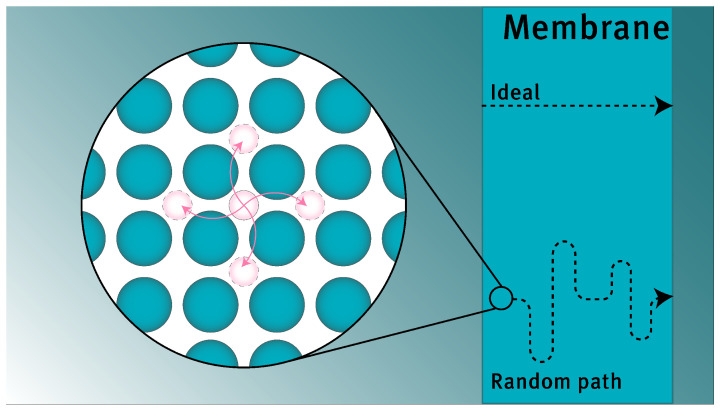
Schematic representation of the equal jumping probability towards all directions, and the resulting random path that the interstitial will travel.

The information available does not allow for every pre-exponential factor to be calculated directly via Equations (10) and (11). Hence, Equation (12) is applied for the dissociative adsorption and Hess’ law is employed to enable the use of relative entropies, the resulting expressions being Equations (13)–(18) [20,27]. The pre-exponential factor for diffusion constitutes parameters related to the diffusive jump distance and unit cell geometry (*a* and $\gamma_{D}$), these are added to relate the rate of diffusion to the jumping frequency [28]. Alternative descriptions for Equation (18) exist which contain a correction factor for the correlation of subsequent jump directions. The literature only provides values for the case of self-diffusion [15]. These cannot be applied since the concentration of diffusing species is far lower in interstitial hydrogen diffusion than in self-diffusion. In addition, the primary diffusion mechanism for hydrogen is not via vacancies. Hence, it would suffice to use a value of one for this parameter and thus omit it [29]. The current modeling framework implies that a diffusive jump has the same probability of occurring in any direction. This results in a diffusion path that is a random walk rather than a straight line from retentate to permeate side, as shown schematically in Figure 2. For subsurface penetration and subsurface egression, it is assumed that the pre-exponential factor is related to diffusion by a factor $\frac{1}{3}$, as only one-third of all diffusive jumps close to the surface lead to the desired transition (being the jump perpendicular to the surface, and assuming that the species is free to jump in three directions) [15].

(12) $$\begin{array}{cc} \Lambda_{1}^{+} & =\frac{p^{0}}{c_{s}}\sqrt{\frac{1}{2\pi M_{H_{2}}RT}} \end{array}$$

(13) $$\begin{array}{cc} \Lambda_{1}^{-} & =\Lambda_{1}^{+}\exp\left(-\frac{\Delta S_{1}}{R}\right) \end{array}$$

(14) $$\begin{array}{cc} \Lambda_{5}^{+} & =\Lambda_{1}^{+}\exp\left(-\frac{\Delta S_{5}}{R}\right) \end{array}$$

(15) $$\begin{array}{cc} \Lambda_{2}^{-} & =\frac{1}{3}\frac{k_{B}T}{h}\exp\left(\frac{\Delta S_{D}^{‡}}{R}\right) \end{array}$$

(16) $$\begin{array}{cc} \Lambda_{2}^{+} & =\Lambda_{2}^{-}\sqrt{\frac{\Lambda_{1}^{-}}{\Lambda_{1}^{+}}}\exp\left(\frac{\Delta S_{S}}{R}\right) \end{array}$$

(17) $$\begin{array}{cc} \Lambda_{4}^{-} & =\Lambda_{2}^{-}\sqrt{\frac{\Lambda_{5}^{-}}{\Lambda_{1}^{+}}}\exp\left(\frac{\Delta S_{S}}{R}\right) \end{array}$$

(18) $$\begin{array}{cc} \Lambda_{D} & =\gamma_{D}a^{2}\frac{k_{B}T}{h}\exp\left(\frac{\Delta S_{D}^{‡}}{R}\right) \end{array}$$

The given equations constitute the framework of the current model. After all chemokinetic parameters have been established, Equations (2)–(6) may be solved for the surface coverages, concentrations and flux, taking into account that the first and fifth equations consider the flux of molecular hydrogen whilst the other equations consider the flux of atomic hydrogen. The set of equations is solved using the Levenberg–Marquardt algorithm, as readily available in Matlab, under the constraint that the time derivative of either the surface coverage or concentration of atomic hydrogen in any portion of the membrane equates to zero, corresponding to the steady-state solution. Although this method has been shown to provide accurate results in the recent literature [30], it is prone to converge to local minima in the phase space, which does not correspond to realistic situations (e.g., negative concentrations). This is due to the sensitivity of the solution method to the initial values. To find the most optimal solution within the Levenberg–Marquardt methodology, i.e., to more thoroughly explore the phase space, *N* parallel trial systems with sufficiently different (randomly generated) initial values were constructed and executed. Any trial systems that converged to values containing negative surface coverages, concentrations or fluxes were obviously discarded. Moreover, cases where all values were positive but $x_{p}>x_{r}$ or $\theta_{p}>\theta_{r}$ were discarded as well, since these also represent unrealistic solutions (in the case where $p_{H_{2,r}}>p_{H_{2,p}}$, that is). Of the remaining solutions, the solution with the lowest residual value was chosen. If the residual value was larger than the tolerance settings of the optimization procedure, the complete procedure was reinitialized using a smaller parameter interval and a higher value of *N*.

For validation of the computational method, the obtained solution was compared to the solution of a more robust method: time-integration. The system of equations was rewritten to differential form and was also time-integrated until the steady-state solution was reached. Herein, the system was initialized with surface coverages and concentrations of zero. The *ode15s* algorithm of Matlab was used, which is a variable step, variable order method based on backward difference formulas for stiff problems [31]. For each of the three metals under investigation, five operating conditions were sampled and tested using this procedure. In all cases, the same result was obtained using both the Levenberg–Marquardt methodology and the direct time-integration of the ordinary differential equations. Hence, the proposed solving strategy based on the Levenberg–Marquardt algorithm with *N* parallel trial systems is validated. It should be emphasized that it could be considered to use time-integration for all calculations in this study. However, while this method is considered to be more robust, solving using the proposed methodology is computationally faster and has the additional advantage that it can trivially be parallellized by the construction of *N* parallel trial systems.

## 3. Parameter Determination

The parameters required for the implementation of the microkinetic model are determined based on the following assumptions. Where applicable, justification of these assumptions is provided in this section.

- Liquids are assumed to be quasi-crystalline, meaning that a crystal structure can be employed in describing the liquid.
- There is no expansion or contraction of the lattice due to hydrogen dissolution.
- There is no interaction between adsorbed and dissolved species amongst themselves.
- The host metal contains no defects, grain boundaries, internal friction or other crystal non-idealities.
- The membrane is modeled as a single phase and phase transitions, such as liquid–solid, α-β in palladium or BCT-FCC in indium, are not taken into account.
- FCC interstitials always occupy octahedral sites whilst BCC interstitials always occupy tetrahedral sites. Only octahedral-octahedral jumps or tetrahedral-tetrahedral jumps are allowed. Only jumps to nearest-interstitial sites are allowed.
- The unit cell is assumed to expand proportionally to the temperature-dependent density.
- Only pure metals can be modeled, as relevant parameters are hard to obtain for alloys.
- The ideal gas law is valid.

The energies required for the modeling of the liquid metals are determined via the Pauling Bond Valence-Modified Morse Potential (PBV-MMP) methodology by Yen et al. [21]. Their broader framework for the determination of activation energies and enthalpies, based on Hess’ law and the works of Shustorovich, is also adopted [15]. One difference in implementation is chosen, namely the expression for the binding energy of a homonuclear diatomic molecule, and the adopted expression is shown in Equation (19). This expression is proposed as its counterpart in the work of Yen et al. seems to have been subject to an error in derivation and the validity of this expression cannot be confirmed through the original UBI-QEP method of Shustorovich and Sellers [32].

(19) $$Q_{H_{2}\cdot M_{2}}=\frac{9D_{H\cdot M}^{2}}{6D_{H\cdot M}+16D_{H\cdot M}}$$

To determine the relevant energies within this framework, the metal-hydrogen binding energy and the coordination numbers of the different binding states are required. The former is taken from the literature and tabulated in Table 2, alongside other relevant parameters. It can be seen that the binding energy of atomic hydrogen is significantly lower on the liquid metals compared to palladium. Using the binding energy of molecular hydrogen (435.7 kJ ${\mathrm{mol}}^{-1}$), a fundamental difference is observed as the dissociative adsorption is exothermic for palladium, but endothermic for the liquid metals. The adsorption enthalpy influences the rate of dissociative adsorption and it can thus be expected that this rate is lower for the liquid metals compared to palladium.

For the relevant coordination numbers, geometric considerations are required, which are also largely based on the work of Yen et al. [15]. In this study, it is assumed that the crystal structure of the solid state can be used to describe a metal in its liquid state (the quasi-crystalline approach). Various works describe the coordination number of the metals as relatively constant when going through the solid–liquid phase transition, which makes this a valid assumption [15,33,34]. Further proof is provided by previous work considering the diffusion of hydrogen in nickel and copper. In these studies, the pre-exponential factor for diffusion is nearly the same for both liquid and solid phases [3,35,36]. The crystal structures to be used are BCO for gallium and BCT for indium [37,38]. The phase diagrams of these metals are rich in different crystal structures, but the structures at standard temperature and pressure will be used here [39]. The former is modeled as BCC where transport through the top and bottom faces of the unit cell is hindered and the latter can be modeled as a distorted FCC structure [39]. The surface adsorption site is in both cases modeled as FCC (111) hollow [15]. These assumptions lead to the coordination numbers shown in Table 2. The influence of crystal geometry on diffusion can be expressed through the parameter $\gamma_{D}$. Values of $\frac{4}{6}$ and $\frac{1}{12}$, are selected for gallium and indium, respectively, [28,40]. As for the diffusive jump distance, this parameter is related to the lattice constant, which is in turn calculated from the temperature-dependent density. The jump distance is adjusted and averaged, accounting for the non-cubic shape of the gallium and indium unit cells [37,38].

This leaves two final structural parameters. Firstly, the saturation solubility of hydrogen, $\chi_{H\cdot M_{s}}$, is obtained by dividing the number of interstitial sites by the number of metal atoms. Secondly, the concentrations of bulk and surface sites can be determined by the temperature-dependent density and the molar mass and diameter of the metal atom, as in Equations (20) and (21), respectively.

(20) $$\begin{array}{cc} c_{b} & =\frac{\rho_{M}}{M_{M}} \end{array}$$

(21) $$\begin{array}{cc} c_{s} & =d_{M}c_{b} \end{array}$$

Using the geometric considerations for the different metals, the liquid free volume can be estimated, again taking into account the non-cubic liquid metal unit cells. With this parameter, the entropy change of dissolution can be approximated. This is again done via the framework of Yen et al. [15]. The free volume theory used, originally proposed by Frank and Evans, was found to fail in the current model for certain cases where lattice expansion due to dissolution was not taken into account [41,42,43]. The volume available for a hydrogen atom was calculated to be negative here and this resulted in anomalous entropies. In these cases, total loss of gas-phase entropy (as calculated by the Shomate equation for molecular hydrogen) was assumed upon dissolution. The framework of Yen et al. was adopted as well for the entropy change associated with diffusion, presenting a new complication for the situation where the model calculates a negative volume for the interstitial site [15]. In this case, being the case where there is zero entropy in the dissolved state, the entropy of diffusion is assumed to be zero, as the absolute entropy cannot be negative.

In considerations regarding the entropy of adsorbed species, a different approach was adopted, assuming constrained two-dimensional motion of adsorbates on the membrane surface. In this approach, the overall adsorption entropy takes the form of Equation (22). The entropy of an adsorbed species is subsequently calculated by subtracting one translational degree of freedom from the gas-phase atomic configurational entropy [44]. As mentioned, there is no complete two-dimensional translational freedom, and it was found by Campbell and Sellers that a factor of 0.68 suffices in describing the correlation between the adsorbed entropy and the gas-phase entropy, as shown in Equation (23) [45]. The required gas-phase atomic configurational entropy can be determined from the Sackur–Tetrode equation, as shown in Equation (24) [44]. The Sackur–Tetrode equation requires the molecular volume in the gas phase (which is determined via the ideal gas law) and the thermal wavelength, as shown in Equations (25) and (26), respectively. For the determination of the entropy contribution of one translational degree of freedom, argon is used as a reference, as shown in Equation (27). The molar mass of this noble gas is 39.95 g ${\mathrm{mol}}^{-1}$ and its reference entropy has a value of 154.6 J ${\mathrm{mol}}^{-1}$ $K^{-1}$ [45,46]. It should be noted that this approach is only valid when assuming that the influence of the vibrational and rotational degrees of freedom on the overall entropy is negligible. This assumption is generally accepted in cases with small molecules and atoms [44].

(22) $$\begin{array}{cc} \Delta S_{i} & =2S_{H,\mathrm{ad}}-S_{H_{2}} \end{array}$$

(23) $$\begin{array}{cc} S_{H,\mathrm{ad}} & =0.68(S_{H}-S_{H,1D}) \end{array}$$

(24) $$\begin{array}{cc} S_{H} & =R\ln\frac{e^{5/2}v_{g}}{\lambda_{t}^{3}} \end{array}$$

(25) $$\begin{array}{cc} v_{g} & =\frac{k_{B}T}{p} \end{array}$$

(26) $$\begin{array}{cc} \lambda_{t} & =\frac{h\sqrt{N_{\mathrm{AV}}}}{\sqrt{2\pi M_{H}k_{B}T}} \end{array}$$

(27) $$\begin{array}{cc} S_{H,1D} & =\frac{1}{3}\left(S_{\mathrm{Ar}}^{0}+R\ln\left({\frac{M_{H}}{M_{\mathrm{Ar}}}}^{3/2}{\frac{T}{298}}^{5/2}\right)\right) \end{array}$$

It was found that the postulated approach provided a high entropy in the adsorbed state (e.g., a lot of freedom for adsorbed species to translate), which is close to the molecular gas-phase entropy (meaning that $\Delta S_{i}$ is close to zero). This can be explained by the fact that, although the adsorbed species are more constrained in their translational freedom, two species can move independently, rather than one species in the gas phase. This may be an acceptable situation in some cases, but likely not in palladium membranes, where surfaces are often full [47]. For refinement of entropy values, Equation (22) was modified by introducing a factor that decreases the entropy as a function of surface coverage, as shown in Equation (28) [45]. This factor only comes into play at coverages higher than 0.5 and lower than unity. These constraints are added as the former condition will provide increased entropy, being counter-effective, and the latter condition ensures that the logarithm of zero is never reached [48].

(28) $$\Delta S_{i}=2S_{H,\mathrm{ad}}-S_{H_{2}}+R\ln\frac{1-\theta_{i}}{\theta_{i}}$$

Whilst including a coverage-dependent term in the determination of parameters does complicate the process to obtain a numerical solution, implementation of Equation (28) results in a more acceptable desorption-limited operating region for palladium. When assuming total loss of entropy upon adsorption, it was predicted that a membrane as thin as 1 μm at a temperature of 373 K would already operate in the diffusion-limited regime, which is unlikely [13,20,49,50]. It should still be stressed that it is very difficult to validate whether or not this approach is justified, as there is virtually no experimental data on the desorption-limited regime available. Nevertheless, it is expected that the use of this coverage-dependent term will aid in discerning the relevant phenomena in liquid metal membranes compared to palladium membranes. This is the case as the surfaces of liquid metal membranes are expected to be relatively empty, leading to the omission of the third term on the right-hand side of Equation (28) and a more favourable entropy of adsorption, compared to palladium membranes, as a result.

## 4. Results and Discussion

### 4.1. Model Validation

**Figure 3:**
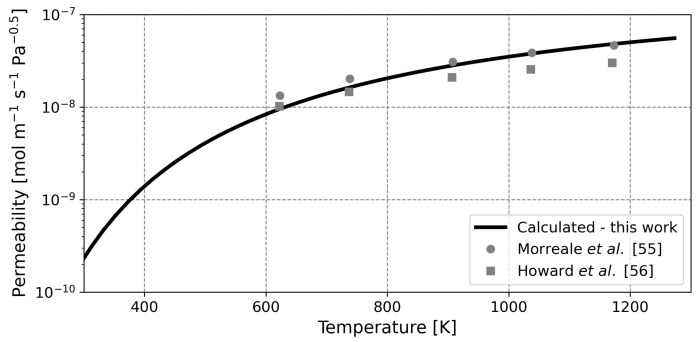
Comparison between calculated and experimentally obtained permeabilities for pure palladium. Different markers represent different studies and the line represents calculations. Experimental data from Morreale et al. and Howard et al. [55,56].

Validation of the current model is complicated by the scarcity of experimental liquid metal membrane data. To overcome this complication, this study uses the broad applicability of the proposed model and taps into the abundance of data available for palladium membranes. For the initial validation, permeability data of experimental cases for self-supporting palladium membranes is used, as shown in Figure 3. Note that the different studies, whilst representing pure palladium membranes, are not exactly in agreement. Discrepancies between the studies may arise due to differences in the fabrication method, crystal defects, surface roughness, pinholes, polycrystallinity and measurement errors [11,42,52,53]. It should be kept in mind that these experimental discrepancies can also influence the validation, as the model does not include numerical analogues to account for these effects.

In order to validate the current model, the permeability of a pure palladium membrane was calculated over a broad range of temperatures. Conventionally, these calculations are done by means of the Sieverts’ constant. Since this parameter is not explicitly defined in the current model, the permeability was calculated via Equation (29). Note that this approach (in the current framework) requires the concentration of hydrogen to be calculated first, after which Equation (29) can be solved. In calculating the concentration of hydrogen, a membrane with a thickness of 10 m was considered, as this ensures that diffusion is truly rate-limiting. Since the PBV-MMP methodology is devised for liquid metals, these validating simulations are conducted with the energies of the hydrogen-palladium system as proposed by Ward and Dao, tabulated in Table 3 [13]. The resulting permeability values are plotted in Figure 3, along with the aforementioned experimental values. The full range of temperatures that is modeled can currently not be achieved experimentally, but it serves to show the applicability of the current model and what can be expected of future membranes, which may be able to operate out of the current operating window of temperature.

(29) $$P_{H_{2}}=\frac{D_{H}c_{b}\chi_{H\cdot M_{s}}x_{r}}{2\sqrt{p_{H_{2},r}}}$$

The calculated values are in good agreement with data from the literature. This shows that the current model is able to adequately describe the hydrogen permeability of palladium membranes as a function of temperature. The calculated activation energy for permeation is fitted with a value of 17.9 kJ ${\mathrm{mol}}^{-1}$ (with a coefficient of determination of 0.992). The fact that the modeled permeability is slightly higher compared to experimental values can be expected, as non-idealities usually lower the permeability [17,54].

**Figure 4:**
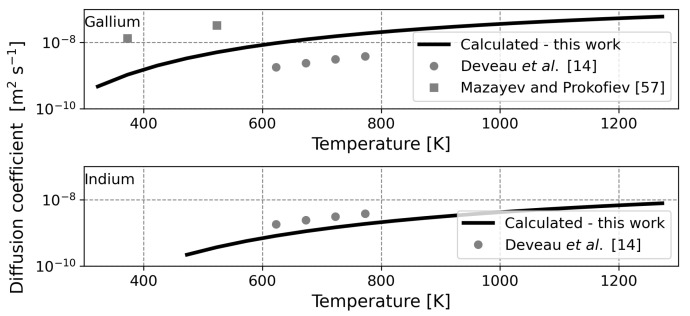
Calculated and experimentally obtained diffusion coefficients for gallium (**top**) and indium (**bottom**). The *X*-axis is shared. Dots represent experiments and lines represent calculations. Data from Mazayev and Prokofiev and Deveau et al. [14,57].

To continue the model validation, the calculated liquid metal diffusion coefficient is compared to experimental data. For gallium, two correlations describing the diffusion coefficient of hydrogen as a function of temperature are available in the literature. The correlations differ quite significantly; the activation energy has a value of 9.6 kJ ${\mathrm{mol}}^{-1}$ in one case and a value of 20 kJ/mol in the other [14,57]. The activation energy as calculated by the current model is 14.94 kJ ${\mathrm{mol}}^{-1}$, which is in between the values from the literature. The comparison between diffusion coefficients is displayed in the top graph of Figure 4. Herein, an adequate coherence is found. Again, the calculated curve does not match either of the two experimental curves, but this is found to be acceptable in the adopted ‘order of magnitude’ approach since the experimental data is not in agreement.

For indium, there is only one source of experimental data available. Calculations for this liquid metal yield an activation energy of 19.2 kJ ${\mathrm{mol}}^{-1}$, a value comparable to the empirical value of 25.7 kJ ${\mathrm{mol}}^{-1}$ [14]. In the lower graph of Figure 4, it is seen that calculated and experimental data are in agreement. This validates the assumption that indium can be modeled using a distorted FCC crystal structure.

For both metals, the hydrogen diffusion coefficient is approximately an order of magnitude higher than the self-diffusion coefficient [58,59]. This is convincing evidence for the use of the quasi-crystalline approach. However, it should be noted that the observed diffusion of hydrogen may, in part, be attributed to self-diffusion of the liquid metal (i.e., due to movement of the unit cell). Nevertheless, despite that the observable value is possibly lumped, the current model is able to replicate empirical values.

**Figure 5:**
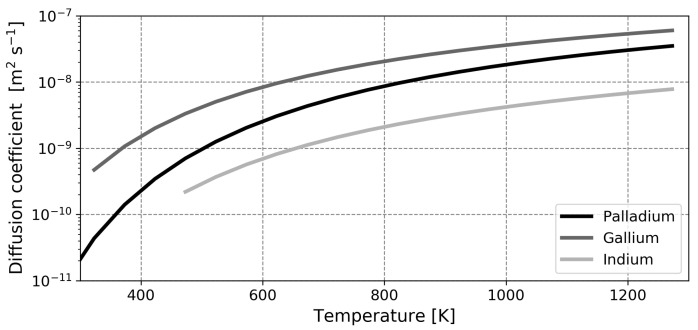
Calculated diffusion coefficients for palladium, gallium and indium.

For a graphic comparison of the diffusion coefficients in the liquid metals relative to the diffusion coefficient in palladium, the calculated values for the three metals are plotted in Figure 5. It is seen that the diffusion coefficient of hydrogen is higher in gallium compared to palladium, whilst the values are lower for indium. This may hint at a higher permeability in gallium, but it should be noted that the saturation solubility, i.e., the relative amount of interstitial sites for hydrogen, is lower in gallium compared to the other two metals [14].

### 4.2. Rate-Determining Step Analysis

**Figure 6:**
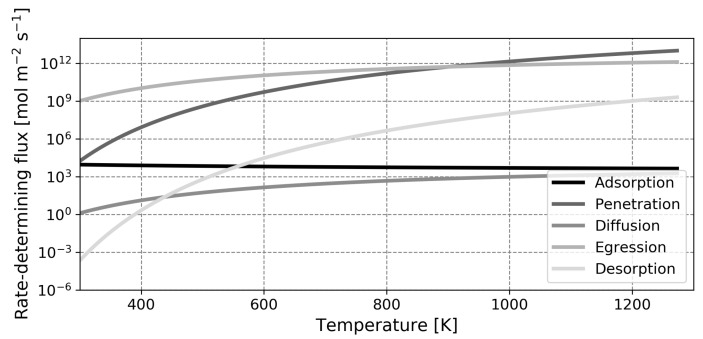
Representation of the hydrogen flux through a palladium membrane for different rate-determining elementary reaction steps. A membrane with a thickness of 1μm was modeled.

**Figure 7:**
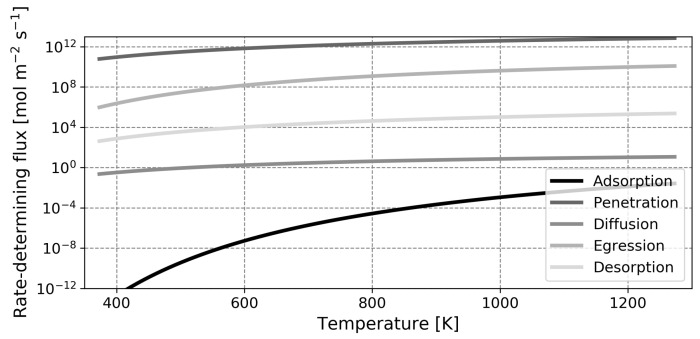
Representation of the hydrogen flux through a gallium membrane for different rate-determining elementary reaction steps. A membrane with a thickness of 100μm was modeled.

**Figure 8:**
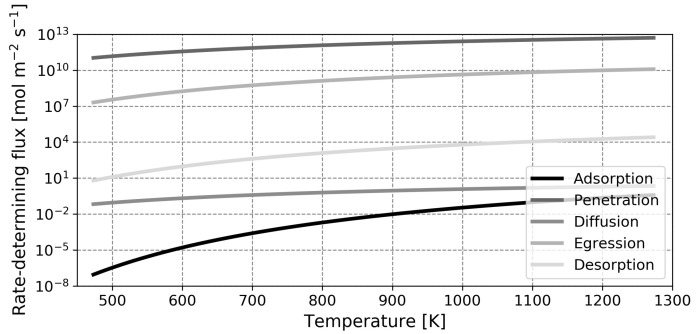
Representation of the hydrogen flux through an indium membrane for different rate-determining elementary reaction steps. A membrane with a thickness of 100μm was modeled.

It is anticipated that the influence of surface phenomena is greater in liquid metal membranes, as there is a significant energetic barrier present for the dissociative adsorption of hydrogen in the two liquid metals under study. In the spirit of the pioneering studies by Ward and Dao, the relative influence of the different steps in the permeation process can be made quantitative through a rate-determining step analysis [13]. In Figure 6, Figure 7 and Figure 8, the flux of atomic hydrogen is plotted for palladium, gallium and indium, in the hypothetical situation where one of the five forward permeation steps is rate-limiting. The calculations were performed with a retentate pressure of 1 atm and a permeate pressure of 0 atm, using a thickness of 1 μm for palladium and thicknesses of 100 μm for gallium and indium. These values were chosen as they represent state-of-the-art thicknesses that are practically feasible for the different metals (for the liquid metals, the high surface tension and difficulty in preparing leak-free films necessitates a relatively high thickness) [12,60]. The implications of this choice in thickness, in the form of a flux analysis, are discussed later. Note that the coverage-dependent entropy factor Equation (28) was determined with a coverage of 0.9999 for palladium and a value of 0.5 for the liquid metals as these values are representative and using values of either zero or one will lead to mathematical complications as the natural logarithm will approach either infinity or negative infinity.

An initial point of interest in the rate-limiting step analysis is the difference in range of the *Y*-axis. It is seen that for the specified thickness and over the temperature range investigated, the rate-limited flux of a gallium membrane spans 12 orders of magnitude whilst the flux of a palladium membrane spans only 9 orders of magnitude. Moreover, it can be seen that for palladium, the relevant steps are desorption and diffusion (which is in agreement with the literature), whilst for gallium and indium, adsorption and diffusion are critical steps [13,50]. Over the temperature range investigated, both liquid metals operate in the adsorption-limited regime and the higher diffusion coefficient of gallium relative to indium does not provide an advantage. As previously mentioned, the low rates of dissociative adsorption of the liquid metals compared to palladium are largely due to the low binding energy of hydrogen on the liquid metal surface. Comparing the adsorption-limited flux at 773 K, values of $5.64\times 10^{3}$ mol $m^{-2}$ $s^{-1}$, $1.43\times 10^{-5}$ mol $m^{-2}$ $s^{-1}$ and $1.19\times 10^{-3}$ mol $m^{-2}$ $s^{-1}$ are obtained for palladium, gallium and indium, respectively. These values follow the differences in binding energy in Table 2 and the higher flux of indium compared to gallium is explained by the lower energetic barrier for dissociative adsorption of this metal. Furthermore, it was already shortly introduced that whilst the diffusion coefficient of hydrogen in gallium is higher than the diffusion coefficient of hydrogen in palladium, the saturation solubility is lower by a factor two in the former. This manifests itself in a higher diffusion-limited flux for palladium.

In this assessment, the diffusion-limited flux was determined in the situation where $x_{r}=1$ and $x_{p}=0$. This is a hypothetical situation which does not occur in reality and hence, the factor $x_{r}\left(1-x_{p}\right)$ will therefore be significantly lower than unity. This means that the influence of diffusion is noticed beyond the strictly diffusion-limited regimes in Figure 6, Figure 7 and Figure 8 and the transition to this regime likely occurs at a lower temperature.

**Figure 9:**
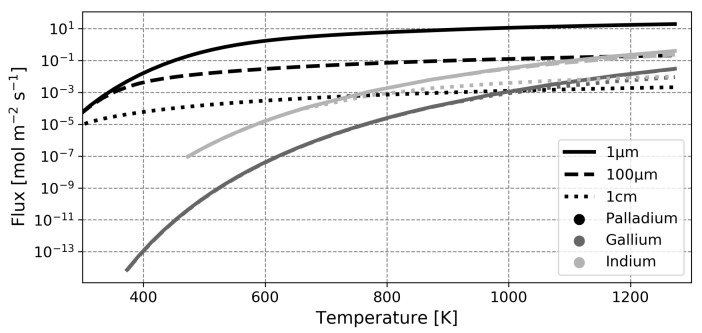
Representation of the hydrogen flux through gallium, indium and palladium membranes of different thicknesses. Calculations were done with a retentate pressure of 1atm and a permeate pressure of 0atm. All curves were interpolated with a coefficient of determination higher than 0.99.

To further investigate whether the diffusion-limited regime may indeed be broader, evaluation of the membrane flux as a function of thickness and temperature is done by full model calculations, shown in Figure 9 for the three metals under study. It was hypothesized through the rate-determining step analysis that gallium and indium operate mostly in the adsorption-limited regime. Hence, the thickness should not significantly influence the membrane flux. For gallium, all lines follow the same adsorption-limited path over nearly the entire temperature range, with the curve for a membrane thickness of 1 cm as the exception, where it starts to deviate slightly at very high temperatures. This confirms the hypothesis that for gallium decreasing the membrane thickness does not increase the flux. For an indium membrane of 1 cm, the flux is governed mostly by adsorption until a temperature of about 873 K. After that, diffusion starts to play a role. For smaller thicknesses (being 100 μm and thinner), however, it is seen that an indium membrane may operate in the adsorption-limited regime over the whole temperature range, in agreement with the hypothesis.

It can be observed that at very high temperatures, indium foils could outperform palladium foils of 100 μm thick. This comparison, however, is unlikely to be relevant, since state-of-the-art palladium-based membranes are only 10 μm or thinner.

### 4.3. Assessment of the Potential of Liquid Metal Membranes

The presented results from the microkinetic modeling study show that palladium will outperform the liquid metals under study in virtually all realistic cases. One of the few remaining advantages of gallium and indium compared to palladium would be the lower price per kilogram. The price of palladium is assumed to be EUR 64,735 per kg (from Johnson Matthey as of October 2021), whereas the price of gallium is EUR 599 per kg and the price of indium is also EUR 599 per kg (both from Rotometals as of October 2021). This means that both metals are 108 times as cheap as palladium. Through Figure 9, it can already be concluded that over the temperature range investigated, a palladium membrane of 1 μm will outperform gallium and indium membranes of 108 μm thick. The remaining strategy would then be to increase membrane area rather than membrane thickness. Using this approach, under the assumption that stable liquid metal membranes of every thickness may be produced, the flow rate through a palladium membrane with a surface area of 1 m${}^{2}$ may be compared to the flow rates through gallium and indium membranes with surface areas of 108 m${}^{2}$. This is done in Table 4 for different thicknesses.

The data in Table 4 shows that indium outperforms gallium for all cases, confirming that indium is indeed the superior liquid metal. Moreover, it is seen that situations exist where an indium membrane permeates more hydrogen than a palladium membrane of the same cost. This occurs only at thicknesses of about 100 μm and larger, which is not a fair representation of the state-of-the-art of supported palladium membranes. In addition, the comparison in Table 4 does not include the costs of support material and membrane production, which account for a sizeable portion of the overall membrane cost, typically 50 to 75% [61]. Finally, the reactor may even have to be oversized to accommodate the large membrane surface areas. These combined factors further decrease the feasibility of the liquid metal membrane concept as the lower cost per kilogram does not necessarily scale to a cheaper membrane process.

This analysis shows that the current concept has a lot in common with the group V metals, more established non-palladium membrane materials. These metals were also praised for their high diffusion coefficient, but their catalytic activity towards the dissociative adsorption of hydrogen is lacking [3,62]. For these materials specifically, this hurdle is overcome through the sandwiching of the metal between thin layers of palladium. Thus, combining the high surface activity of palladium with the high permeability of the group V metal [63,64]. In applying this strategy to liquid metals, intermetallic diffusion and alloying are likely to be detrimental to the concept, given the extremely high reactivity of gallium and indium towards other metals [65,66]. A sandwiched configuration will therefore likely not be stable.

## 5. Conclusions

Based on the results from this microkinetic modeling study, it can be concluded that liquid metal membranes for hydrogen separation based on gallium or indium will likely operate in the adsorption-limited regime. Hence, optimization of these membranes cannot be achieved through a reduction of membrane thickness. This allows for thicker, more stable, membranes, but it was shown that these liquid metal membranes will underperform supported thin-film palladium-based membranes in all realistic cases, even when accounting for the much lower cost of the liquid metals. The application of the concept of liquid metal membranes is therefore seriously hampered, similarly to membranes based on group V metals, which also lack sufficient catalytic activity for the dissociation of hydrogen. Especially when taking into account the experimental complications that were experienced in producing thin liquid metal membranes that are stable under relevant gas atmospheres, it is to be questioned whether the proposed liquid metal membranes are ever a feasible alternative for conventional palladium-based membranes.

## Figures and Tables

**Table 1:** Elementary reaction steps of the five-step permeation process [15]. *S* represents a surface site and *X* represents a bulk site, while subscripts *r* and *p* represent the retentate and permeate side, respectively.

Step	Reaction
1	H_2_,r +2S${}_{r}$ ⇌ 2H · S${}_{r}$
2	H · S${}_{r}$ + X${}_{r}$ ⇌ H · X${}_{r}$ + S${}_{r}$
3	H · X${}_{r}$ + X${}_{p}$ ⇌ H · X${}_{p}$ + X${}_{r}$
4	H · X${}_{p}$ + S${}_{p}$ ⇌ H · S${}_{p}$ + X${}_{p}$
5	2H · S${}_{p}$ ⇌ H_2_${}_{,p}$ +2S${}_{p}$
Overall	H_2_,r ⇌ H_2_${}_{,p}$

**Table 2:** Overview of the relevant parameters in the modeling of membranes based on palladium, gallium and indium [13,15,19,37,38,51].

Parameter	Palladium	Gallium	Indium
Crystal structure	FCC	BCO	BCT
$D_{H\cdot M}$	259 kJ ${\mathrm{mol}}^{-1}$	166 kJ ${\mathrm{mol}}^{-1}$	192 kJ ${\mathrm{mol}}^{-1}$
$d_{M}$	278 pm	244 pm	284 pm
$n_{s}$	3	3	3
$n_{s}^{‡}$	2	2	2
$n_{b}$	6	10	6
$n_{b}^{‡}$	4	4	3
$\chi_{H\cdot M_{s}}$	1	0.5	1
c/a	1	1.69	1.53
$\gamma_{D}$	1/12	4/6	1/12

**Table 3:** Energetic parameters used for the modeling of palladium membranes, as proposed by Ward and Dao [13].

Parameter	Value	Unit
$\Delta H_{S}$	−8.368	$kJ\;{\mathrm{mol}}^{-1}$
$E_{1}^{+}$	0	$kJ\;{\mathrm{mol}}^{-1}$
$E_{1}^{-}$	83.00	$kJ\;{\mathrm{mol}}^{-1}$
$E_{2}^{+}$	55.65	$kJ\;{\mathrm{mol}}^{-1}$
$E_{2}^{-}$	22.18	$kJ\;{\mathrm{mol}}^{-1}$
$E_{D}$	22.18	$kJ\;{\mathrm{mol}}^{-1}$

**Table 4:** Calculated flow rates through palladium, gallium and indium membranes of the same metal cost as a function of membrane thickness. All values calculated at 773K, 1atm retentate pressure and 0atm permeate pressure.

Thickness	$\Phi_{\mathrm{Pd},1m^{2}}$	$\Phi_{\mathrm{Ga},108m^{2}}$	$\Phi_{\mathrm{In},108m^{2}}$
1 μm	5.28 mol $s^{-1}$	$1.30\times 10^{-3}$ mol $s^{-1}$	$1.21\times 10^{-1}$ mol $s^{-1}$
10 μm	$6.27\times 10^{-1}$ mol $s^{-1}$	$1.30\times 10^{-3}$ mol $s^{-1}$	$1.21\times 10^{-1}$ mol $s^{-1}$
100 μm	$6.48\times 10^{-2}$ mol $s^{-1}$	$1.30\times 10^{-3}$ mol $s^{-1}$	$1.21\times 10^{-1}$ mol $s^{-1}$
1 mm	$6.52\times 10^{-3}$ mol $s^{-1}$	$1.30\times 10^{-3}$ mol $s^{-1}$	$1.14\times 10^{-1}$ mol $s^{-1}$
1 cm	$6.53\times 10^{-4}$ mol $s^{-1}$	$1.30\times 10^{-3}$ mol $s^{-1}$	$6.82\times 10^{-2}$ mol $s^{-1}$

## Data Availability

The Matlab model used to generate the data presented in this study is available on request from the corresponding author.

## Abbreviations

The following abbreviations are used in this manuscript:

BCC	Body-Centered Cubic
BCO	Body-Centered Orthorhombic
BCT	Body-Centered Tetragonal
FCC	Face-Centered Cubic
PBV-MMP	Pauling Bond Valence-Modified Morse Potential
UBI-QEP	Unity Bond Index-Quadratic Exponential Potential

## Nomenclature

**Symbol**	**Representation**
*a*	Lattice constant
*c*	Unit cell height
$c_{b}$	Concentration of bulk sites
$c_{s}$	Concentration of surface sites
$D_{H}$	Diffusion coefficient of hydrogen
$D_{H\cdot H}$	Binding energy of molecular hydrogen
$D_{H\cdot M}$	Hydrogen-metal binding energy
*d*	Diameter
*E*	Activation energy
*H*	Enthalpy
*h*	Planck constant
*J*	Flux
*K*	Solubility
*k*	Reaction rate constant
$k_{B}$	Boltzmann constant
*M*	Molar mass
*N*	Solver sample size
$N_{\mathrm{AV}}$	Avogadro constant
*n*	Coordination number
*P*	Membrane permeability
*p*	Pressure
$Q_{H_{2}\cdot M_{2}}$	Binding energy of molecularly adsorbed hydrogen
*R*	Gas constant
*r*	Reaction rate
*S*	Entropy
*T*	Temperature
$v_{g}$	Atomic volume
*x*	Bulk concentration
$\gamma_{D}$	Structural factor for diffusion
δ	Membrane thickness
θ	Surface coverage
Λ	Arrhenius pre-exponential factor
$\lambda_{t}$	Thermal wavelength
ρ	Density
Φ	Molar flow rate
$\chi_{H\cdot M_{s}}$	Saturation solubility

## Subscripts and Superscripts

**Subscript**	**Representation**
1D	One-dimensional
ad	Adsorbed
b	bulk
D	Diffusion
H	Atomic hydrogen
H_2_	Molecular hydrogen
p	Permeate
r	Retentate
S	Solution
s	Surface
0	Reference
+	Forward
−	Backward
‡	Transition state
